# The impact of electronic cigarettes on pregnancy and childhood health outcomes: the ECHO study—a protocol for a multicentre, prospective, observational, cohort

**DOI:** 10.1007/s00404-025-08066-8

**Published:** 2025-06-20

**Authors:** Eibhlín F. Healy, Abigail O’Connell, Marah Shaikh Yousef, Adele Reddin, Michael Boyle, Tim Coleman, Anne Doolan, Patricia Fitzpatrick, Kate Frazer, Shane Higgins, Cecily Kelleher, Fergal D. Malone, Eoin O’Curráin, Ricardo Seguardo, Jennifer Walsh, Des Cox, Michelle Downes, Carmen Regan

**Affiliations:** 1 The Coombe Hospital, Dublin, Ireland; 2 The Royal College of Surgeons in Ireland, Dublin, Ireland; 3 University College Dublin, Dublin, Ireland; 4 The Rotunda Hospital, Dublin, Ireland; 5 The University of Nottingham, Nottingham, UK; 6 The National Maternity Hospital, Dublin, Ireland; 7 Children’s Health Ireland, Dublin, Ireland

**Keywords:** Vaping, Smoking cessation in pregnancy, Preterm birth

## Abstract

**Background:**

Stopping cigarette smoking in pregnancy positively impacts on the incidence of preterm birth, intrauterine growth restriction, and stillbirth. Electronic cigarettes (E-cigarettes) are viewed by some women as a lower risk alternative to tobacco smoking during pregnancy and are cautiously endorsed in some parts of the UK to support smoking cessation(1), however, robust, high-quality data on the impact of E-cigarettes on pregnancy and childhood health outcomes are lacking.

**Objective:**

The objective of the ECHO study is to investigate the impact of maternal E-cigarette use during pregnancy on obstetric, neonatal, and infant outcomes.

**Methods:**

Pregnant women attending three, large, standalone, urban, university, maternity units in Dublin, Ireland, will be invited to take part in this prospective, multicentre, observational cohort. Smoking and vaping patterns will be assessed using digital patient questionnaires. Objective assessment of breath carbon monoxide and urine cotinine levels will accurately determine exposure status at pre-specified timepoints. A third trimester ultrasound will assess growth and fetal dopplers across all groups. Postnatally, infants will undergo anthropometric measurements and developmental checks. A subgroup of 150 randomly selected infants will be assessed with the Bayley’s Scales of Infant and Toddler Development.

**Discussion:**

Nicotine-containing E-cigarettes are addictive and their long-term impact on pulmonary, cardiovascular, and neurological function on developing humans is undetermined. This study is ambitious as it aims to longitudinally assess the outcomes of the mother/baby dyad, examining the impact of vaping on pregnancy, neonatal and infant health, growth and neurocognitive outcomes.

**Trial registration:**

ClincialTrials.gov NCT06297005

## Background

Combustible cigarette smoking is the foremost modifiable risk factor for adverse pregnancy outcomes [2]; therefore, encouraging women to stop smoking in pregnancy is a key public health priority [3]. Combustible cigarette smoking during pregnancy is associated with an increased risk of fetal anomaly [4], small for gestational age and stillbirth, preterm birth, low birth weight [5], placental abruption, sudden infant death syndrome [6], respiratory morbidity in the neonatal period [7], asthma and longer term neurodevelopmental delay [8]. Increasingly, it is accepted that intrauterine growth and development are strong determinants of health, not only in the neonatal period, but also impact on childhood and adult health outcomes [9, 10]. Pregnancy can be a motivator for smoking cessation, with quit attempts higher than at any other time in a woman’s life [11]; smoking cessation prior to or during the first trimester of pregnancy significantly reduces the risk of preterm birth [8] and impacts positively on birthweight [12].

Nicotine replacement therapy (NRT) in the form of patches, inhalers, lozenges etc., has been prescribed for pregnant women seeking pharmacological support to stop smoking for decades; it accepted to be significantly less harmful to the fetus than continuing to smoke combustible tobacco cigarettes [13, 14].

Electronic cigarettes (E-cigarettes) or ‘vapes’ are viewed as a reasonable alternative to combustible cigarette smoking by some women and are now endorsed by some health policy makers in the United Kingdom to support women to stop cigarette smoking in pregnancy [15]. However, there is a paucity of high-quality, prospective data assessing the impact of electronic cigarettes on pregnancy, neonatal, and infant outcomes. In the following protocol, registered with ClincialTrials.gov (NCT06297005), we outline a multicentre, prospective, observational cohort study of pregnancies exposed to vaping to address this research gap.

### Why is combustible cigarette smoking cessation during pregnancy so important for adverse birth outcomes?

Globally, preterm birth is the leading cause of neonatal morbidity and mortality [16]; in addition to the significant personal toll, the economic impact of preterm birth cannot be overstated [17]. Complete combustible cigarette smoking cessation during pregnancy could reduce preterm birth rates in high-income countries by 5–8% for extreme preterm birth (<32 weeks), 3–4% of preterm births (<37 weeks), and crucially a 5–7% reduction in stillbirth [18, 19]. Supporting smoking cessation during pregnancy can positively impact maternal, fetal, infant, child, and therefore overall population health in the longer term; however, the optimum means to facilitate smoking cessation in pregnancy is undetermined [12] and there are high rates of relapse in the puerperium.

### Nicotine and nicotine replacement therapy

Nicotine is a naturally occurring alkaloid and the addictive component of tobacco and nicotine-containing vape products. Although previous publications advised of the teratogenic potential of nicotine, [20] their conclusions are largely extrapolated from studies of pregnant combustible cigarette smokers or from exposed animal models [7, 21]. A recent, large, multi-national, retrospective, cohort study robustly demonstrated that there was no increased risk of major congenital malformation in women using NRT in the 90 days prior to and during pregnancy [14]. The compelling question remains regarding the issue of neurocognitive impact in infants of mothers exposed to nicotine: evidence here is sparse.

During pregnancy, nicotine metabolism is enhanced in comparison to the non-pregnant population [22]. The effectiveness of NRT in assisting cigarette smokers to stop smoking has not been replicated in the pregnant population. This is potentially due to sub-therapeutic dosing levels related both to the quitter’s concerns about nicotine exposure [23] and the prescribers’ lack of training [24]. Nicotine readily crosses the placental barrier and there is evidence of accumulation of nicotine in fetal serum and amniotic fluid in higher concentrations than in maternal serum [25]. In addition, neonates demonstrate a diminished ability to metabolise nicotine, resulting in a half-life of three to four times that of an adult for second-hand smoke [2]. NRT, although not first line, is conservatively endorsed internationally to aid supporting women to stop cigarette smoking in pregnancy [26]. Whilst there is no robust evidence to state that NRT causes significant harm during human pregnancies [13, 14], its effectiveness for smoking cessation in this cohort is mixed [27].

### Electronic cigarettes/vapes

Electronic cigarettes, colloquially known as ‘vapes’, are battery powered devices that heat a solution (e-liquid) to create a nicotine-containing aerosol. In addition, the e-liquid often contains different flavourings, as well as the compounds propylene glycol and glycerine. The evaporation of the liquid at the heating element is followed by rapid cooling to form an aerosol which is directly inhaled (or “vaped”) by the user through a mouthpiece. Each device includes a battery, a reservoir that contains the liquid, and a vaporisation chamber with a heating element [28]. The newest generation E-cigarette devices facilitate blood serum nicotine concentrations that are equivalent to that of a traditional combustible cigarette [29]; the method of use is similar to conventional combustible cigarettes resulting in an experience for the user that is closer to cigarette smoking than any other forms of NRT, and likely explains the growing popularity of vapes as a smoking cessation tool [30]. Importantly, vaping does not generate carbon monoxide, the primary, deleterious by-product of combustible cigarette smoking.

### Note on nomenclature

Electronic cigarette users will be identified as ‘vapers’ throughout the manuscript; combustible cigarette smokers as ‘smokers’; and women who use neither electronic nor combustible cigarettes will be identified as controls.

## Objectives

The ECHO study has four primary objectives described below:

- The primary obstetric objective is to establish whether infants born to women who vape are at a higher or lower risk of adverse outcomes in pregnancy compared to infants of combustible cigarette smokers and non-smokers.
- The primary neonatal objective is to assess anthropomorphic variation, if any, in newborn babies born to vapers in comparison to smokers and controls. The primary paediatric objective is to establish whether parent-reported and doctor-confirmed wheeze and/or asthma occurs more frequently in children exposed to vaping in utero compared with children of combustible cigarette smokers and non-smokers.
- The primary neurocognitive objective is to assess if there are significant differences in neurocognitive outcomes between the infants of a women who vape compared to infants of women who used combustible cigarette smokers and non-smokers.
- Globally, we aim to correlate, obstetric, neonatal, pulmonary, growth and neurocognitive differences, if any, to quantitative levels of cotinine excreted in maternal urine at in the third trimester.

### Proposed study design—PEOCT framework

**Fig. 1:**
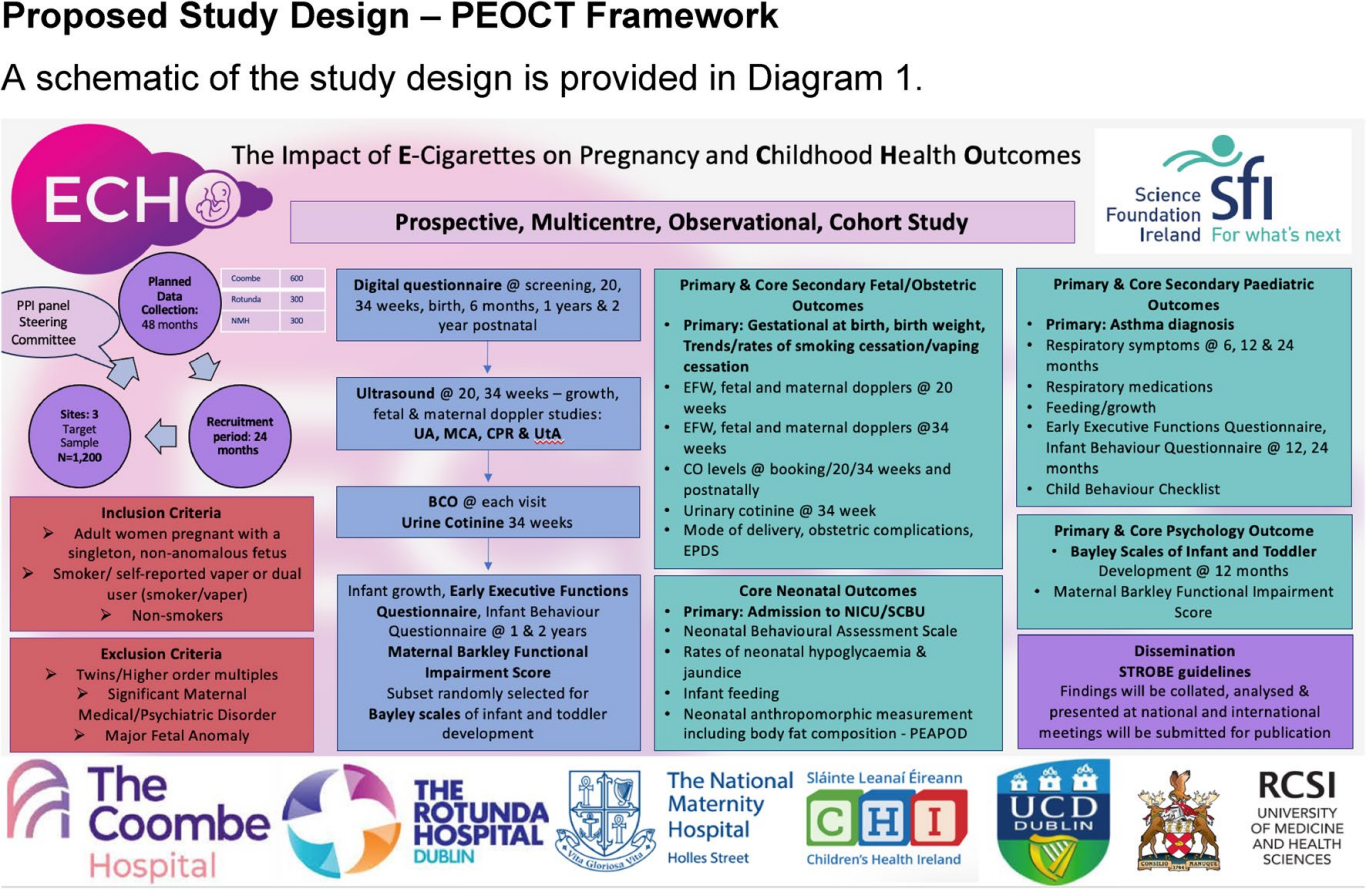
Study schematic

A schematic of the study design is provided in Fig 1

### Population

This prospective, multicentre, observational cohort will focus on women pregnant with a singleton fetus who self-report vaping, cigarette smoking, vaping and cigarettes smoking, or no exposure (controls). The obstetric portion of the study operates from three recruitment sites; The Coombe Hospital, The Rotunda Hospital, and the National Maternity Hospital—these standalone maternity units, based in Dublin, Ireland’s capital city, cumulatively deliver approximately 22,000 women annually [31–34].

Smoking and vaping patterns will be assessed using a combination of digital validated patient questionnaires administered at set timepoints throughout pregnancy, in addition to objective assessment of exhaled breath carbon monoxide (BCO) assessed by point of care exhaled carbon monoxide breathalysers (Bedfont’s ‘Smokerlyzer’® device) at each antenatal study visit. Nicotine levels will be assessed using quantitative urinary cotinine, analysed by Eurofins Biomnis enzyme immunoassay.

Women are recruited prior to 24-week gestation after being directly asked about smoking and vaping status in a structured fashion at their booking visit; if they fulfil the eligibility criteria, they are then invited to partake in the study as either a vaper, a smoker, a smoker/vaper or a control (Table 1.).

**Table 1:** Exposure status

Vaper	Vaper/smoker	Smoker	Control
• Reports vape use • BCO ≤4 ppm	• Reports dual use • BCO ≥ 5 ppm	• Reports cigarette smoking • BCO ≥ 5 ppm	• Denies cigarette smoking or vaping • BCO ≤4 ppm

A BCO measurement of ≥ 5 parts per million (ppm) was selected to define active smoking, in keeping with Irish National Clinical Guidance. Selection of BCO ≥ 5 ppm as a cutoff confers a sensitivity of 0.9 and specificity of 0.92 [35]. This is an important consideration in accurately classifying both smokers and smoker/vapers in addition to self-reported exposure status.

After screening, demographic and biometric data points are gathered, and a series of questionnaires are administered digitally as per the schedule outlined below:

### Sequence of questionnaire timepoints

- At recruitment to assess baseline cigarette smoking and vaping history
- After 20 weeks/mid-trimester
- Third trimester
- After birth
- Aged 6 months
- Aged 1 year
- Aged 2 years

Self-administered, digital questionnaires, accessed on a smartphone, laptop or PC, are completed by participants without the presence of an interviewer; this may increase the validity of the reported exposure, hence the decision to prefer this method [36] when gathering data on frequency, duration and reasons for vaping. Questionnaires included validated instruments (Fagerstrom) to assess nicotine dependence in both cigarette smokers and vapers [37, 38]. Infants of study participants in exposure and control groups will be assessed at 12 and 24 months corrected for gestational age at birth.

### Exposure

The exposure of interest is electronic cigarette use, referred to as ‘vapes’. Nicotine exposure is objectively determined by sampling urine cotinine in the third trimester. Urine cotinine is a metabolite of nicotine, and accepted, reliable reflection of recent nicotine use; it is the most widely used biomarker for the metabolism of nicotine in the human body with 70–80% of nicotine being converted to cotinine in the liver, of which 10–15% is excreted in the urine; it is a highly specific and sensitive marker for nicotine use in the short term [39]. Cotinine has a longer half-life in comparison to nicotine (19 versus 2 h), and therefore is helpful in determining nicotine use over the course of days rather than hours in the case of BCO [40].

### Inclusion criteria

- Women aged 18 or greater and less than 46 years of age at booking.
- Pregnant with a singleton fetus.
- Smoker or vaper or smoker/vaper or control.
- Able to understand and read English.
- Willing to agree to follow-up for the 2-year duration of the study in the postnatal period

### Exclusion criteria

- Twins or higher order multiples
- Late bookers—defined as booking after 24+0 weeks’ gestational age
- Fetus or infant diagnosed with a major congenital abnormality requiring immediate admission to NICU +/- outward transfer for surgery, chromosomal abnormalities
- Any inherited disorder of metabolism or cystic fibrosis on Guthrie card
- History of significant maternal medical disorder
- History of significant maternal psychiatric disorder, e.g. delusional or psychotic disorders, use of >1 psychotropic drugs for treatment
- Serious co-morbid addiction issues, e.g. opiate abuse
- Severe intellectual disability or lack of capacity

## Outcomes

### Primary outcomes

The primary outcomes outlined in Table 2 have informed the sample size calculation. As the outcomes for the postnatal psychological assessments are exploratory, we have used a pragmatic sample target of 150.

**Table 2.** Primary outcomes

Primary outcomes		
Obstetrics/neonatal	Paediatrics	Psychology
• Preterm birth • Low birth weight	• Doctor-diagnosed wheeze	• Neonatal Behavioural Assessment Scale • Bayley’s Infant and Toddler Development (*n*=150)

### Obstetric secondary outcomes

- Trends in smoking cessation and crossover from smoking to vaping and from vaping to non-smoking/non-vaping/smoking.
- BCO at screening, 20 weeks, third trimester, and postnatally.
- Quantitative urine cotinine assay in the third trimester.
- Estimated fetal weight (EFW)—using the Hadlock formula [41]—fetal and maternal dopplers at 20 weeks and 32–34+6 (maternal: uterine artery Doppler, fetal: umbilical artery, middle cerebral artery, cerebroplacental ratio).
- Incidence of small for gestational age (SGA) defined as EFW and/or AC less than the 10th centile for gestational age on ultrasound at any gestation after study entry.
- Diagnosis of congenital malformation.
- Birth centile [42].
- Blood pressure measurement at screening, 20 weeks and third trimester, and postnatally.
- Incidence of essential hypertension—high blood pressure at booking defined at a BP 140/90 mmHg.
- Incidence of diagnosis of pregnancy-induced hypertension and incidence pre-eclampsia [43].
- Incidence of pre-labour premature rupture of membranes (PPROM).
- Incidence of anaemia, defined as a haemoglobin level of less than 10g/dL at booking, 28 weeks or any other time point in the antenatal period.
- Maternal history of abnormal cervical screening cytology or cervical surgery.
- Indication for and incidence of induction of labour.
- Mode of delivery.
- Delivery complications.
- Incidence of postnatal caesarean, episiotomy or perineal wound infection requiring antibiotics.
- Edinburgh Postnatal Depression Scores prior to discharge.
- Incidence of venous thromboembolic (VTE) disease—new onset deep venous thrombosis or pulmonary embolus.
- Unscheduled admission to hospital.
- Admission to high dependency unit (HDU) or inter-hospital transfer to intensive care unit.

### Secondary neonatal outcomes

- APGAR scores at 1 and 5 minutes.
- Brazelton’s Neonatal Behavioural Assessment Scale (NBAS).
- Umbilical artery blood gas (if available).
- Admission to neonatal intensive care (NICU) or special care baby unit (SCBU).
- Incidence of neonatal hypoglycaemia as per local guidelines.
- Incidence of neonatal jaundice requiring phototherapy.
- Mode and type of infant feeding.
- Neonatal anthropometric measurements (weight in grams, length, head, chest and waist circumference in centimetres).
- A subgroup of 150 infants will have PEAPOD assessment. The PEAPOD is a specialised scale that both weighs a baby and uses air displacement plethysmography to determine the baby’s body volume. These measurements allow us to calculate the baby’s density and estimate the baby’s body fat and lean body mass [41].

### Paediatric secondary outcomes

- Paediatric anthropometric measurements (weight, height, body mass index, head circumference, waist circumference, chest circumference and mid-arm circumference measurements) at 12 and 24 months.
- Episodes of wheeze, bronchiolitis, pneumonia and ENT infections and/or wheeze at 6, 12 and 24 months.
- Frequency, timing and intensity of wheezing episodes.
- Prescribed respiratory medications.
- Diagnosis of eczema.
- Number of attendances/admissions to hospital.

### Neurocognitive and psychological secondary outcomes

- Early Executive Functions Questionnaire (EEFQ) at 12 and 24 months.
- Infant Behaviour Questionnaire (IBQ) at 12 months.
- Child Behaviour Checklist (CBCL) at 24 months.
- A subgroup of 150 infants will have a Bayley Scales of Infant and Toddler Development assessment performed at 12 months of age.
- *Maternal* Barkley Functional Impairment Score at infant 1 year visit.
- Edinburgh Postnatal Depression Score at birth and penultimate study visit.

### Study duration

The anticipated duration of the study is 48 months, with an active recruitment period of 15–18 months.

### Sample size and planned statistical analyses

We will aim to achieve as representative as sample as possible of each population. Descriptive statistics on outcomes and other risk factors will be derived for the three groups at each timepoint. An assessment of representativeness of each group will be made based on baseline indicators. In the case that key measurements are missing at rates in excess of 5%, multiple imputation procedures will be considered. Longitudinal analyses of the outcomes will be conducted using (normal or logistic) multilevel (mixed effects) models to account for repeated measurements within individuals. Generalised additive models (GAMs) or logistic GAMs (lGAM) will be used to estimate group differences in each outcome over time in a fully flexible model that can allow for non-linear change without overfitting. Potential confounders or modifiers of the outcomes will be incorporated into the models as predictors or by utilising interaction terms as appropriate.

### Power calculations and rationale

The hypotheses of the ECHO study are that:

**Table:** 

Women who vape during pregnancy will be more likely have a preterm birth and that their infants will have a lower birth weight (LBW), lower neuro-behavioural scores and increased respiratory symptoms when compared with non-smokers.
Women who vape during pregnancy will be less likely to have a preterm birth and their infants will have a higher birth weight, higher neuro-behavioural scores, and fewer respiratory symptoms than when compared with tobacco smokers.

The best available data suggest a prevalence of smoking rate during pregnancy in Ireland is between 9 and 10% [44], whilst the current expected rate of vaping during pregnancy is approximately 5% [45]. The cumulative live birth rates in the three maternity hospitals in Dublin in 2023 were: The Coombe Hospital—6974, The Rotunda Hospital—8442, and the National Maternity Hospital—6880, which will give a projected denominator of approximately 22,000 total live births. The incidence of vaping during pregnancy in the Coombe is 5% [34, 46]; projected to the other three hospitals gives approximately 750–1250 vapers per annum. The calculations in Table 3 give the magnitude of a detectable association with (2-year) prevalence of each condition (odds ratios) or mean differences that are detectable at a power of 80%, with a type I error rate of 0.05. These assume use of simple uncorrected Chi-square test or *t* tests. Detectable effect sizes are presented assuming a range of sample sizes to illustrate how power is maintained under a range of recruitment scenarios. We plan to collect three equally sized groups—non-smokers, smokers, and vapers

**Table 3:** Sample size calculation

Sample size (per group)	Baseline incidence		
	10% (wheeze)	6.2% (preterm birth)	5% (low birth weight)
	Detectable odds ratios:		
100	3.00	3.62	3.19
200	2.25	2.63	2.26
300	1.96	2.24	1.91
**400**	**1.80**	**2.04**	**1.74**

Whilst robust data on the effect of vaping during pregnancy on women and infants are lacking, the effects of smoking are well characterised on neonatal anthropometric measurements. Asthma or wheeze has an incidence of approximately 10% within the first 3 years of life [47]. LBW occurs at a frequency of about 5% [48]. PTB occurs in approximately 6.2% of births in Ireland [49]. Maternal smoking has been shown to increase risk of asthma or wheeze in childhood with an odds ratio of 1.8 (95% CI 1.35–2.53). Smoking has also been shown to increase incidence of LBW with an odds ratio of 1.75 (95% CI 1.42–2.10). The odds ratio for PTB conferred by smoking is 1.27 (95% CI 1.20–1.33).

Data on neurocognitive outcomes is lacking; neurocognitive outcomes between groups can be expressed as standardised mean differences (SMD). Table 4 gives indicative detectable differences for each sample size. For reference, caregiver depression has been as a risk factor on the NBAS with a SMD of 0.28 [50] and smoking demonstrated an association of the order of SMD = 0.2–0.4 for executive function including inhibition and self-control [51].

**Table 4:** Indicative detectable differences per sample size

Sample size (per group)	Standardised mean difference
100	0.40
200	0.28
300	0.23
**400**	**0.20**

Therefore, we propose to recruit 1200 participants (400 smokers, 400 vapers and 400 non-smoker, non-vaper controls) to generate adequate power and precision to answer our research questions.

In the case that key measurements are missing at rates in excess of 5%, multiple imputation procedures will be considered. We anticipate the likelihood of missing data is small given the standard procedures in place to manage the study centrally.

Case report form (CRF) data entry into REDCap will include extensive data validation checks. Missing data will be monitored, and strategies will be developed to minimise its occurrence. Central statistical data monitoring will summarise missing or inconsistent data periodically. The study workbook will be approved by the chief investigator and validations will also be made that will cross-check the study workbook with the ECHO study electronic CRF.

### Demographic and baseline disease characteristics

Demographic and baseline disease characteristic data will be summarised for each treatment group by presenting frequency distributions and/or descriptive statistics.

Age, parity, medical and surgical history, social history, drug and allergen history, previous obstetric and gynaecological history will be collected from the booking assessment which will be part of the source documentation.

### Data monitoring

Study participants data are anonymised—each participant is assigned a sequential number and site identifier. All study data will be entered into the REDCap storage electronic database by trained staff with restricted access and multilevel password protection. Data management support will be through the Clinical Research Centre at University College Dublin. A data management plan has been formulated by the Chief Investigator and data manager to comply with GDPR regulations and will follow the FAIR data principles. Data generated from this study will be used for the purposes of this study only and not shared with people or organisations outside this group.

### Evaluation of adverse and serious adverse events

Adverse events (AE) and serious adverse events (SAEs) associated with vaping, specifically the development of E-cigarette/vape-associated lung injury (EVALI) will be reported to the study steering committee and to the sponsor, but they will not trigger cessation of the study due to its observational nature. The specific AE events and SAEs broadly adhere to the Maternal and Fetal Adverse Event Terminology Delphi consensus guidance [49], but also include unscheduled admission to hospital, admission to a high dependency unit (HDU) or transfer out to an intensive care unit (ICU), maternal death, the diagnosis of a venous thromboembolism in the mother, the diagnosis of e-cigarette or vaping associate pulmonary injury (EVALI) in mother or child, the death of a child.

### Public patient involvement—PPI

A PPI panel was assembled including the primary stakeholders, stop smoking charities, smoking cessation midwives, pregnant women who identify as vapers, smokers, and ex-smokers, contributed to and reviewed the study design. These contributors have agreed to remain on a panel to provide further review on any relevant subsequent study-specific collaborations.

### Ethics

The ECHO study has been approved by the Research Ethics Committees (RECs) of the Coombe Hospital, the Rotunda Hospital, and the National Maternity Hospital, in addition to UCD. This study may be terminated at the request of the Chief Investigator, REC, or other regulatory authority if, during the study, concerns about participant safety, data integrity emerge, or poor recruitment occur. This study will be conducted in accordance with Good Clinical Practice (GCP), as defined by the International Conference on Harmonisation (ICH) and in accordance with the ethical principles underlying European Union Directive 2001/20/EC and 2005/28/EC.

### Dissemination

The study will be reported adhering to the STROBE guidelines for the reporting of observational trials [49]. The findings will be collated, analysed, and presented at national and international meetings in oral and poster format, and will be submitted as scientific papers to journals for publication. We anticipate the findings of the ECHO study will inform national and international guidelines on vaping and smoking in pregnancy.

## Discussion

Vaping is an exposure that is undeniably increasing. The approach currently adopted in some parts of the UK is endorsement of vaping as a method of harm reduction in pregnancy; this pragmatic approach is occurring in a vacuum of evidence around safety, and challenges the advice that the medical community have historically offered to pregnant people—which is to abstain from potentially harmful exposures during gestation. Nicotine-containing vapes are addictive and their long-term impact on pulmonary, cardiovascular and neurological function in both adult and developing humans is undetermined. This study is ambitious as it aims to longitudinally assess the outcomes of the mother and baby dyad, examining not only the impact of vaping on pregnancy, but also on neonatal and infant health outcomes. A major strength of this study is the sequential assessment of breath carbon monoxide and urine cotinine to provide objective clarification of outcomes in relation to each exposure—previous large registry studies [52] have demonstrated higher levels of SGA and LBW in vapers; however, these data are drawn from post hoc questionnaires, and are subject to recall and selection bias. A prospective, observational cohort from The Coombe Hospital in Dublin found no difference in the incidence of SGA and LBW in vapers versus controls [46]. In addition, the robustly planned paediatric and neurocognitive assessment of exposed versus non-exposed infant will assist women, their midwives and doctors, to make informed health choices during pregnancy in the future.

## Data Availability

No datasets were generated or analysed during the current study.
